# Role of miRNAs in Bovine Oocyte Maturation and Reproductive Regulation

**DOI:** 10.3390/ijms26072828

**Published:** 2025-03-21

**Authors:** Xiaogeng Yang, Honghong He, Peng Wang, Yaying Wang, Linlin Wang, Falong Yang, Jian Li, Huizhu Zhang

**Affiliations:** 1 Key Laboratory of Animal Medicine, Southwest Minzu University of Sichuan Province, Chengdu 610041, China; xgengy@126.com (X.Y.); honghong3h@126.com (H.H.); yayingwang666@163.com (Y.W.); falong.yang@swun.edu.cn (F.Y.); jianli_1967@163.com (J.L.); 2 Key Laboratory of Qinghai-Tibetan Plateau Animal Genetic Resource Reservation and Utilization, Ministry of Education, Chengdu 610041, China; 3 Animal Husbandry Science Institute of Ganzi Tibetan Autonomous Prefecture, Kangding 626000, China; roc1213@163.com; 4 College of Pharmacy and Food, Southwest Minzu University, Chengdu 610041, China; jiayouwl123@163.com

**Keywords:** miRNA, *bovine*, oocyte maturation, embryo development, ART

## Abstract

MicroRNAs (miRNAs) are a class of endogenous small non-coding RNAs that regulate target gene expression in many eukaryotes. MiRNAs are essential for post-transcriptional regulation, influencing various biological functions, including oocyte growth and maturation, fertilization, early embryo development, and implantation. In recent decades, numerous studies have identified a substantial number of miRNAs associated with mammalian oocyte maturation and early embryo development, utilizing methods such as small RNA sequencing and modulating miRNA expression through overexpression or inhibition. In this review, we introduce the biosynthesis of miRNAs and their regulatory roles in germ cells, summarizing the expression patterns and post-transcriptional regulation of miRNAs during *bovine* oocyte maturation and early embryo development, as well as their potential application in *bovine* assisted reproductive technology (ART).

## 1. Introduction

*Bovine* is an important agricultural species, playing a central role in meat and dairy production. With the development of in vitro maturation (IVM), in vitro fertilization (IVF), and in vitro embryo production technologies, the study of *bovine* oocyte and embryo development has significant implications for improving reproductive efficiency, enhancing embryo quality, promoting bioengineering technology, increasing economic benefits, and fostering fundamental basic scientific research.

Oocyte maturation is essential for successful embryo development. Research indicates that oocyte maturation is mainly divided into nuclear maturation and cytoplasmic maturation [1]. Enhancing the quality and maturity of oocytes can significantly increase the rate of embryo development [2]. However, embryo survival and development remain major challenges in IVF and in vitro embryo production techniques. MicroRNAs (miRNAs) have emerged in recent years as potent regulators of gene expression that are widely expressed in biological processes such as cell growth, development, differentiation, proliferation, apoptosis, cell death, and signaling [3,4,5], which are critical for oocyte maturation and the normal progression of embryo development. In the context of mammalian reproduction, miRNAs play an important role in sex differentiation [6], gametogenesis, fertilization, early embryonic development [7], and pregnancy [8] within the context of mammalian reproduction. Research focusing on miRNAs during *bovine* follicular development has shown that these molecules are crucial for regulating follicular development [9,10,11]. Notably, studies conducted by Zielak et al. have shown that the levels of miRNAs in follicular fluid fluctuate throughout the stages of follicular development in *bovine* ovaries [12], suggesting that miRNAs may play a critical role in modulating *bovine* oocyte growth. Furthermore, differential expression of miRNAs has been associated with granulosa cells in preovulatory dominant and subordinate follicles in cattle [13]. Numerous studies have elucidated the dynamics of miRNA expression throughout mammalian germ cell and early embryonic development [14,15,16]. Despite the temporal correlation between differential miRNA expression and oocyte maturation and early embryonic development, our understanding of the specific roles of miRNAs in *bovine* oocyte maturation and embryonic development remains limited. This paper aims to review the biosynthesis of miRNAs and their regulatory roles in germ cells and summarize the expression patterns and post-transcriptional regulation of miRNAs during *bovine* oocyte maturation and early embryo development and their potential application in *bovine* assisted reproductive technology (ART), thus providing a theoretical basis for improving embryo quality and reproductive efficiency in *cattle*.

## 2. Biosynthesis of miRNAs

MiRNAs are endogenous, small, single-stranded non-coding RNA consisting of 18 to 25 nucleotides [17]. MiRNAs regulate gene expression in various eukaryotic organisms and were first discovered in *Caenorhabditis elegans* by Victor Ambro’s laboratory in 1993 [18], at the same time as Gary Rufkun’s laboratory identified the first microRNA target gene [19]. Together, these two landmark discoveries identified a novel mechanism of post-transcriptional gene regulation.

**Figure 1:**
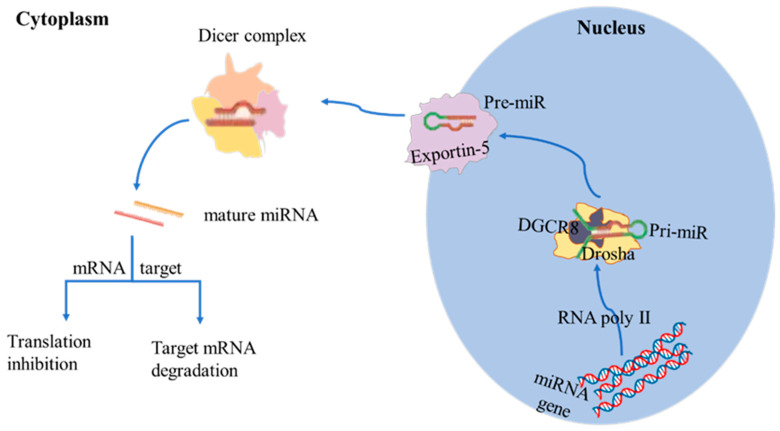
Canonical pathway of miRNAs biosynthesis. Within the nucleus, miRNAs are initially transcribed by RNA polymerase II into pri-miRNAs. The enzyme Drosha forms microprocessor complexes with DGCR8, cleaving pri-miRNA to produce pre-miRNAs. The complexes cleave the pri-miRNA into pre-miRNAs, which are transported to the cytoplasm by Exportin-5. In the cytoplasm, the Dicer complex cleaves the pre-miRNAs, leading to mature miRNAs. The mature miRNAs then interfere with the translation mechanisms by binding to complementary target sequences in the 3′-untranslated region (3′-UTR) of the mRNA, leading to translation inhibition or mRNA degradation.

The biosynthesis of miRNAs involves a series of steps that can occur either inter- or intragenic (Figure 1). MiRNAs are processed through a canonical or non-canonical pathway. The canonical pathway is the one most commonly used pathway during miRNA biogenesis [20]. In the nucleus, miRNAs are first transcribed by RNA polymerase II into primary miRNAs (pri-miRNAs), which can be thousands of nucleotides long and typically have hairpin structures [21]. The enzyme double-stranded RNA-specific endoribonuclease (Drosha) forms microprocessor complexes with DiGeorge critical region 8 (DGCR8) and cleaves pri-miRNA to produce precursor miRNAs (pre-miRNAs) that are approximately 70 nucleotides long [22,23]. Pre-miRNAs are then transported to the cytoplasm by Exportin-5 and further processed by the Dicer to produce mature miRNAs [4]. Unlike messenger RNA (mRNA), miRNAs do not encode proteins; instead, they inhibit or modify protein production by binding to complementary target sequences in the 3′-untranslated region (3′-UTR) of mRNA, thereby interfering with translation mechanisms, resulting in mRNA silencing or degradation [22,24].

## 3. Regulation of miRNAs in Germ Cells

MiRNAs modulate post-transcriptional gene expression by binding to specific mRNA target sequences, mainly through translational inhibition or mRNA degradation [8,25]. During germ cell development, miRNAs have been found to regulate proliferation and differentiation. For example, in male germ cell development, miRNAs are recognized as important regulators of mammalian spermatogenesis, influencing sperm development and maturation by regulating gene expression [26,27]. Several studies have demonstrated the expression of miRNAs at different stages of spermatogenesis, including in sperm cells, spermatocytes, and spermatogonia [28]. Dysregulation of miRNAs has been associated with disorders in sperm production, which subsequently affect fertility [26]. For example, the expression patterns of miR-15a, miR-29b, miR-202-5p, miR-10a, miR-34a, miR-34b, and miR-34c in sperm cells are associated with male fertility in *cattle*, *humans*, and *mice* [29,30,31,32]. The miR-34/449 family plays a crucial role in spermatogenesis and the regulation of spermatozoa maturation and function. In particular, miR-34b/c and miR-449 are highly expressed in postmitotic male germ cells. A deficiency in miR-34bc/449 disrupts both meiosis and the final stages of sperm maturation [33]. In previous studies, the miR-34a knockout *zebrafish* showed a significant increase in progressive sperm motility that is one of the pivotal factors influencing in vitro fertilization rates [34]. In addition, miRNAs play vital roles in the female reproductive system, including follicle development, oocyte maturation, early embryo development, and cell proliferation and apoptosis [35,36]. Current evidence suggests that miRNAs are key molecules involved in the regulation of mRNA translation and degradation during oocyte and early embryo development [14]. The role of miRNAs in reproduction is increasingly being investigated, particularly with regards to their functions and effects on embryo quality [37,38]. Research on miRNAs typically involves targeting Dicer to inhibit miRNA activity. It has been shown that knockout of Dicer1 can result in infertility due to various defects, including an abnormal estrous cycle and atypical responses to gonadotropins, which can lead to irregular ovulation [39]. In addition, the knockout of oocyte-specific Dicer has been shown to prevent the completion of oocyte meiosis by affecting spindle formation and the chromosome structure [40].

### 3.1. Expression Patterns of miRNAs During Bovine Oocyte Maturation

The precise regulation of gene expression is crucial for oocyte development, as changes in expression patterns can result in suboptimal oocyte quality [41]. Research has identified up to 821 genes that are differentially regulated during *bovine* oocyte maturation [42]. MiRNAs, which are short non-coding RNA molecules, play an important role in various biological functions and are regulated in a tissue- and developmental-specific manner [43]. These miRNAs have emerged as key regulators of oocyte maturation, influencing gene expression in a temporally and spatially specific manner [44]. In addition, a variety of organelles are regulated during the stages of oocyte growth, and post-transcriptional regulation is also involved [45]. Research has shown that transcript levels fluctuate over time [42], and this dynamic change may be mediated by miRNAs [9].

**Figure 2:**
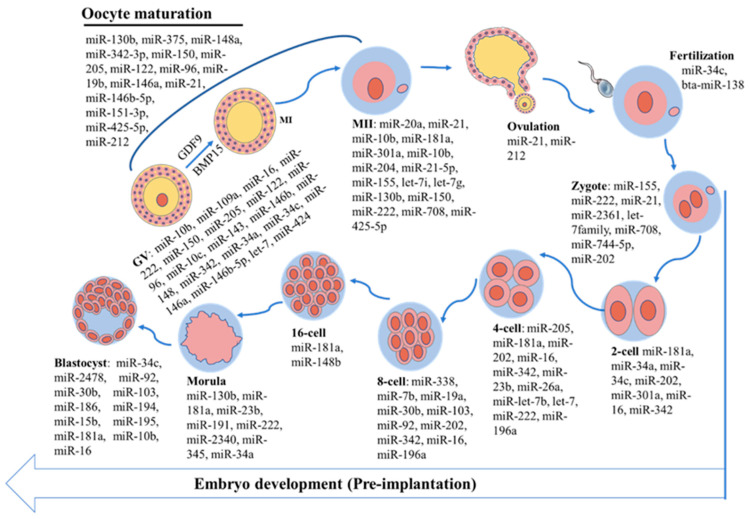
MiRNAs regulate *bovine* oocyte maturation and preimplantation embryo development. GV: germinal vesicle; MI: metaphase I; MII: metaphase II.

During oocyte maturation, miRNAs are involved in the complex molecular processes that ensure successful fertilization and embryo development. Oocyte maturation is characterized by significant changes in gene expression, with specific patterns of miRNA expression being critical. For example, miRNA expression differs between immature and mature *bovine* oocytes. Tesfaye et al. [9] investigated the expression of miRNAs in immature and in vitro mature *bovine* oocytes using a heterologous method. The results showed the differential expression of 59 miRNAs, of which 31 and 28 miRNAs were preferentially expressed in immature and mature oocytes, respectively. This suggests that certain types of miRNAs involved in *bovine* oocyte maturation exhibit fluctuations in expression. Our team established miRNA expression profiles during *yak* oocyte maturation using high-throughput microRNA sequencing technology [46]. We found that 75 miRNAs were differentially expressed in oocytes at the germinal vesicle (GV) and metaphase II (MII) stages; 47 miRNAs were upregulated, and 28 miRNAs were downregulated in the MII oocytes compared to the GV stage. Among the upregulated miRNAs, miR-342 showed the largest fold change (9.25-fold). Similarly, Gilchrist et al. [47] characterized the differential expression of bta-miR-155, bta-miR-222, bta-miR-21, bta-let-7d, bta-let-7i, and bta-miR-190a in *bovine* GV oocytes and MII oocytes. Notably, it was found that pri-miR-155 and pri-miR-222 were not detected in GV oocytes, whereas pri-miR-155 was present in MII oocytes, indicating transcription during maturation. In contrast, the pri-let-7d levels decrease during the maturation process, suggesting that the observed increase in let-7d expression may be attributed to the processing of primary transcripts. These studies demonstrate that both dynamic and stable miRNA populations are present in *bovine* oocytes. This dynamic regulation of miRNAs is essential for the proper timing and coordination of gene expression required for oocyte competence and subsequent fertilization (Figure 2).

### 3.2. Role of miRNAs in Bovine Oocyte Maturation

Oocyte maturation is the process by which oocytes undergo morphological, physiological, and developmental transitions from prophase I to MII [48]. The preovulatory GV oocyte remains arrested in prophase I until a gonadotropin surge triggers nuclear and cytoplasmic maturation events. The oocyte then resumes meiosis through germinal vesicle breakdown (GVBD), extrusion of the first polar body, and chromatin remodeling and reorganization of cytoplasmic organelles and is then arrested again in MII when the oocyte is fertile [47].

MiRNAs play an important role in *bovine* oocyte maturation by regulating the expression of specific genes (Table 1). Andreas et al. [49] investigated the function of miR-20a during *bovine* oocyte maturation by regulating the expression of miR-20a in cumulus-oocyte complexes (COCs) during IVM. The results showed that overexpression of miR-20a could improve the oocyte maturation rate and regulate the expression of oocyte maturation-related genes during IVM. Sinha et al. [50] also found that the inhibition of miR-130b significantly reduced the proportion of oocytes that ejected the first polar body, reached the MII stage, and promoted mitochondrial activity in MII oocytes. More interestingly, miR-130b was also involved in oocyte maturation by regulating *SMAD5* and *mitogen- and stress-activated protein kinase 1* (*MSK1*) genes. The results of Xiong et al. [51] showed that miR-3423p has the potential to promote oocyte maturation of oocytes by targeting the 3′-UTR of *DNA methyltransferase 1* (*DNMT1*) to reduce *DNMT1* mRNA expression. In addition, miR-155mRNA targets *inositol 5-phosphatase 1* (*INPP5D*) [52], and a decrease in *INPP5D* has been shown to increase *AKT* activity [53], a pathway involved in *bovine* oocyte maturation [54]. Similarly, MiR-375 has also been shown to target *bone morphogenetic protein receptor 2* (*BMPR2*) in cumulus cells, thereby affecting cumulus cell proliferation and apoptosis and regulating *bone morphogenetic protein 15* (*BMP15*)/*growth differentiation factor 9* (*GDF9*) receptor expression [55]. Zhang et al. also found that miR-375 regulates oocyte maturation in vitro by targeting *a disintegrin and metalloproteinase with thrombospondin-like motifs 1* (*ADAMTS1*) and *progesterone receptor* (*PGR*) in *bovine* cumulus cells [56]. These results suggest that miRNAs play an important role in regulating oocyte maturation in *bovine* oocytes. Moreover, miRNAs are involved in the regulation of maternal transcripts that are crucial for early embryonic development. During the maternal–embryonic transition (MET), miRNAs facilitate the degradation of maternal mRNAs, allowing for the activation of the embryonic genome. This transition is vital for the successful progression of embryonic development, as it marks the shift from reliance on maternal factors to the activation of zygotic transcription [57].

### 3.3. Expression Patterns of miRNAs in Early Bovine Embryo Development

Mammalian embryonic development begins with the fertilized oocyte, which undergoes continuous division to form an embryo capable of implantation. Early embryonic development depends mainly on maternal mRNA and proteins synthesized during gametogenesis. After fertilization, the embryonic genome is activated (occurs at 8–16 cell stages in *bovines*) [73,74], and the embryonic cells differentiate into the inner cell mass and trophectoderm to facilitate early embryonic developmental processes [75]. Studies have shown that miRNA regulation plays an important role in maternal transcript degradation, embryonic genome activation, and embryonic development during early embryonic development. These critical early developmental processes are controlled and regulated by genes that are expressed in a stage-specific manner [76].

In *bovine* embryos, some miRNAs are stage-specific: miR-125a and miR-496 are specific for four cells, whereas miR-127 and miR-145 are specific for eight cells [8]. However, most miRNAs showed fluctuating expression patterns, depending on the developmental stage of the *bovine* embryo [9]. Paulson et al. [77] sequenced miRNAs using oocytes and preimplantation embryos (1-cell, 2-cell, 4-cell, 8-cell, 16-cell, morula, and blastocyst) to determine the temporal patterns of miRNA expression during *bovine* preimplantation development. The results showed that miRNA expression in embryos was first detected at the two-cell stage but increased significantly at the morula and blastocyst stages. MiR-25 expression increases from the 16-cell stage to the blastocyst stage [9], while miR-181a expression increases early in development and then decreases to low levels in the blastocyst [78]. In addition, Mondou et al. [16] also found an increased expression of miR-130a and miR-21 between fertilized oocyte and 8-cell embryos and were involved in maternal mRNA degradation and gene expression regulation required for embryonic genome activation. However, while miRNAs are expressed at specific stages to regulate embryonic development, it is worth noting that some miRNAs show little or no change between developmental stages and that these miRNAs may play a housekeeping role during critical developmental processes.

### 3.4. Role of miRNAs in Early Bovine Embryo Development

MiRNAs are present in transcriptionally quiescent mature oocytes and preimplantation embryos, showing low levels of transcription before embryonic genome activation. Several studies have shown that embryonic miRNAs are essential for the development of *bovine* preimplantation embryos to the blastocyst stage. miR-208, miR-127, miR-145, and miR-496 [9,15] and miR-135a, miR-218, miR-335, and miR-449b are upregulated in early blastocysts following genomic activation in *bovine* embryos [79]. Sinha et al. [50] analyzed the expression pattern of miR-130b in embryos at different preimplantation stages, and there was no significant change in miR-130b expression levels from zygote to the eight-cell stage. However, significantly increased miR-130b expression was detected at the morula and blastocyst embryo stages. Later, the inhibition of miR-130b expression was found to reduce morula and blastocyst formation by a microinjection of miR-130b inhibitors at the zygote stage. Thus, embryonic miRNAs may regulate embryonic transcription from zygotic gene activation (EGA) and most extensively during the morula-to-blastocyst transition.

MiRNAs have been shown to play important regulatory roles (Table 2) in a variety of biological processes, including cell proliferation, differentiation, apoptosis, and embryonic development [80,81]. *p53* is a target of the let7 family and is known to be involved in cell cycle checkpoints [82] and is present in early *bovine* embryos [83]. Bta-miR-665 improves *bovine* blastocyst development by influencing the microtubule dynamics and apoptosis [84]. In addition, the miRNAs of *bovine* oviduct can also improve embryo development. Cañón et al. [85] demonstrated that microRNA-148b, secreted from extracellular vesicles in the *bovine* oviduct, can improve embryo quality through the *BPM*/*TGF*-β pathway. Similarly, miR-17-5p in *bovine* oviduct fluid (OF) significantly improved embryo development to the blastocyst stage [86]. Aoki et al. [86] identified miRNAs in *bovine* OF and investigated the effect of miR-17-5p in OF on embryo development to the blastocyst stage by the exogenous addition of miR-17-5p mimics. Subsequently, Aoki et al. added miR-151-3p or miR-425-5p to the IVM or IVC medium [70], and these results suggest that miRNAs can promote embryo development to the blastocyst stage. It was also found that eight miRNAs (bta-miR-126-5p, bta-miR-129, bta-miR-140, bta-miR-188, bta-miR-219, bta-miR-345-3p, bta-miR-4523, and bta-miR-760-3p) were upregulated in pregnant cows. By regulating miRNAs in oviductal epithelial cells (OECs) and extracellular vesicles (EVs) in pregnant cows, tubal receptivity can be increased to allow proper embryonic development, leading to a successful pregnancy [87].

On the other hand, the expression patterns of certain miRNAs may also indicate abnormal embryonic development. For example, Pavani et al. [88] isolated 69 differentially expressed miRNAs in EVs from a *bovine* embryo conditioned medium and found that miR-146b showed higher expression in EVs derived from a non-blastocyst (non-developing embryos) embryo conditioned medium. This suggests that miRNA-146b negatively affects the quality and development of *bovine* embryos [88]. Furthermore, increased miR-24 expression correlates with a decreased blastocyst yield [89]. Thus, inhibiting the expression of miRNAs with deleterious effects promotes embryo development and reduces the rate of embryo abnormalities.

**Table 2:** MiRNAs associated with early embryonic development in *bovines*.

MiRNAs	Function	References
bta-miR-183	Regulates microvilli formation and improve early embryonic development by targeting *EZRIN*	[90]
miR-29b	Regulates the expression of *Dnmt3a/3b* and *Dnmt1* in *bovine* SCNT embryos	[91]
miR-34c	Modulates blastocyst quality	[92]
bta-miR-301a	Influences cleavage time and blastocyst formation rate of early embryos by targeting *ACVR1*	[93]
miR-151-3p, miR-425-5p	Improved embryonic development to the blastocyst stage.	[70]
miR-202	Targets *SEPT7* and regulates first cleavage of *bovine* embryos via cytoskeletal remodeling	[94]
miR-449b	Improves the first cleavage division, epigenetic reprogramming and apoptotic of SCNT embryos in *bovine*	[95]

## 4. Potential Applications of miRNAs in *Bovine* ART

MiRNAs have emerged as important regulators of gene expression and have considerable potential in the application of *bovine* ART. The application of miRNAs includes various aspects (Table 3), such as improving the quality and development of *bovine* embryos, sex determination, etc.

### 4.1. Improving Embryo Quality and Development

Although ART has been optimized in several mammalian species, in vitro production (IVP) embryo development is still inferior to in vivo development (IVD) [96]. In *cattle*, approximately 90% of in vitro cultured oocytes undergo nuclear and cytoplasmic maturation, 80% of which are fertilized and divide at least once [97], although only 30% to 40% of oocytes reach the blastocyst stage [96], and pregnancy rates following IVP blastocyst transfer in heifers have been demonstrated to be approximately 40–50% [98]. Therefore, in vitro culture (IVC) conditions are being improved to obtain more blastocysts of higher quality that can continue to develop and implant after embryo transfer and produce viable and healthy individuals.

Several studies have developed different embryo culture systems, such as a coculture with *bovine* oviductal epithelial cells (BOECs) [99] or continuous culture with the addition of oviductal cells and uterine fluid to the medium and the use of extracellular vesicles [100]. Studies have shown that the exogenous addition or suppression of specific miRNA expression can promote healthy embryo development and improve embryo survival and implantation success [50,86,101]. The efficiency of embryo development in ART can be improved by regulating the expression of miRNA. For instance, specific miRNAs have been shown to influence the timing of cleavage and the overall developmental competence of embryos, which are critical factors for successful implantation and pregnancy outcomes [5,102,103].

### 4.2. MiRNAs as Biomarkers in Bovine ART

The expression of miRNAs during *bovine* oocyte maturation and embryo development is tissue- and stage-specific [104], suggesting that miRNAs may act as biomarkers of *bovine* reproductive activities. For example, vesicular miRNAs from follicular fluid are being investigated for their suitability as biomarkers of oocyte quality in mature and immature oocytes [10], as well as healthy and unhealthy follicles [105,106]. This suggests that miRNAs may be important tools for assessing oocyte quality and distinguishing healthy from unhealthy follicles. Similarly, the expression of miRNAs in culture media has been correlated with embryo quality, suggesting that they may serve as non-invasive biomarkers for selecting high-quality embryos during IVF procedures. For instance, specific miRNAs are differentially expressed in the culture media of embryos that successfully develop to the blastocyst stage compared to those that do not. This information can be used to select embryos with a higher likelihood of successful implantation and development, thereby improving the success rates of ART [5,89,102]. The identification of miRNAs as biomarkers for embryo viability can significantly improve the selection process in ART. By analyzing the miRNA profiles in embryos, practitioners can make informed decisions about which embryos to transfer, thereby increasing the chances of successful pregnancies. This approach not only improves reproductive efficiency but also reduces the costs associated with unsuccessful transfers and the management of non-viable embryos. As our understanding of miRNA functions continues to grow, miRNAs will lead to more effective and sustainable practices in *cattle* production. The ongoing research into miRNA profiles and their regulatory roles will be crucial in realizing these potential applications shortly.

### 4.3. Sex Determination

One of the most promising applications of miRNAs in *bovine* ART is in sex determination. The ability to select the sex of offspring is crucial for optimizing breeding programs, particularly in dairy and beef *cattle*. Recent studies have shown that the expression of some miRNAs is sex-specific [8]. For example, miR-124 plays an important role in female gonadal commitment by inhibiting *Sox-9* expression [107], and the deletion of miR-124 prior to sexual commitment can lead to sex reversal in females. miR-202 not only has gonad-specific effects but may also play an important role in preventing premature ovarian failure (POF) [108]. This could allow embryos to be selected on the basis of their sex prior to implantation, thereby increasing the efficiency of breeding programs and ensuring that the desired sex ratio is achieved in livestock production.

**Table 3:** Application of miRNA in ART.

MiRNAs	Function	Application	Species	References
bta-miR-140, bta-miR-92a, bta-miR-222, bta-miR-2285a	Negative association with blastocyst development	Improve embryonic development potential by adding miRNA inhibitors.	*Bovine*	[109]
miR-320a	High expression in high-quality embryo culture medium as a marker of embryo quality	For embryo quality assessment and selection	*Human*	[110]
miR-124	miR-124 regulates *Sox-9* gene affects embryo sex determination	MiRNA-mediated sex-regulation technology to improve sex-specific embryo productivity	*Mice*	[107]
miR-34c	Positive association with embryonic development	Use miR-34c as an indicator of IVF success	*Human*	[111]
miR-210	Inhibit cell migration and trophoblast invasion and angiogenesis	Monitor pregnancy with pre-eclampsia.	*Human*	[112]
miR-155	Negative association with embryonic development	Add miRNA-155 inhibitors to enhance embryonic development	*Porcine*	[113]
Let-7 family, miR-106a	relate genes for regulating oocyte maturation and embryo development	optimize conditions for oocyte maturation and embryo development	*Bovine*	[114]
miR-21	Regulate cell proliferation and apoptosis, improve embryo survival rate	optimize embryo culture conditions and improving embryo quality	*Bovine*	[115]

## 5. Application of miRNA in ART: Limitations and Solutions

As demonstrated in this review, although the application of miRNAs in ART shows great promise, there are a number of limitations and challenges that must be addressed to realize their full potential. The main limitation is the variability in miRNA expression due to individual differences. Factors such as age, health status, and the use of hormone therapy can affect miRNA levels and their function, leading to inconsistent results when miRNAs are used as biomarkers for embryo selection or implantation success. In addition, there is a lack of standardized methods for assessing miRNA expression profiles. For example, miRNA expression levels may vary depending on the sample collection methods, RNA extraction protocols, and analytical techniques used [116]. Another problem is that miRNAs may exhibit off-target effects, as they may bind to unintended mRNA targets, resulting in undesirable alterations in gene expression [104].

There are several strategic solutions to these limitations: (1) using highly sensitive detection methods combined with standardized sample collection, processing, and analysis protocols; (2) performing thorough validation; (3) using well-defined methods to assess miRNA expression in different sample types; and (4) integrating non-invasive miRNA biomarkers with embryo and morphological assessments to improve the embryo selection process [117,118].

## 6. Conclusions

In conclusion, miRNAs are essential regulators of gene expression during *bovine* oocyte maturation and preimplantation embryo development. Their dynamic expression patterns and involvement in critical developmental processes highlight their importance in reproductive biology. Understanding the specific functions and mechanisms of miRNAs in this context could lead to advances in reproductive biotechnology and improved outcomes in *bovine* breeding programs. In addition, miRNAs are likely to serve as molecular markers for assessing embryo developmental competence and sex identification in *bovine* ART in the future. Focusing on the functional characterization of specific miRNAs will provide deeper insights into their role in *bovine* reproduction and may pave the way for innovative strategies to enhance fertility and embryo quality in livestock production.

## Figures and Tables

**Table 1:** MiRNAs associated with oocyte maturation in *bovines*.

MiRNAs	Function	References
miR-302d	Regulates DNA damage and steroid hormone secretion in *bovine* cumulus cells by targeting *CDKN1A*	[58]
miR-375	Proliferation and apoptosis in cumulus cells and oocyte maturation	[56,59]
miR-21-3p	Inhibits *bovine* granulosa cell autophagy	[60]
miR-183-96-182 cluster	Promote *bovine* granulosa cells proliferation and cell cycle transition	[61]
miR-128-3p	Targets *TFEB* and *FoxO4* and activates *bovine* granulosa cells autophagy	[62]
mir-17-92 cluster	regulates proliferation and differentiation of *bovine* granulosa cells	[63]
miR-21	Prevented apoptosis via the *PI3K/Akt* signaling in *bovine* cumulus cells	[64]
miR-125b	Regulates apoptosis by targeting *BMPR1B* in *yak* granulosa cells	[65]
miR-31 miR-143	Regulate steroid hormone synthesis and inhibit cell apoptosis in granulosa cells by targeting *FSHR* gene	[66]
miR-424/503	*Bovine* granulosa cell proliferation and cell cycle progression	[67]
miR-145, miR-125b, miR-199a-3p	Involved in follicle-luteal transition	[68]
miR-424, miR-10b	Abundant in GV oocytes’ role in zygotic genome activation	[69]
miR-212, miR-151-3p, miR-425-5p	Regulation of oocyte maturation	[70,71]
miR-19b	increased the diameter, acetylation levels, and fertilization ability of the oocytes	[72]
